# The Role of Surgical and Perioperative Factors in Shaping Gut Microbiome Recovery After Colorectal Surgery †

**DOI:** 10.3390/antibiotics14090881

**Published:** 2025-08-31

**Authors:** Julia Kohn, Alexander Troester, Zachary Ziegert, Julia Frebault, Sonja Boatman, Maria Martell, Harika Nalluri-Butz, Matthew C. Bobel, Paolo Goffredo, Abigail J. Johnson, Cyrus Jahansouz, Christopher Staley, Wolfgang B. Gaertner

**Affiliations:** 1 Department of Surgery, University of Minnesota, Minneapolis, MN 55455, USAtroes012@umn.edu (A.T.); ziege101@umn.edu (Z.Z.); freba004@umn.edu (J.F.); boat0022@umn.edu (S.B.); cmstaley@umn.edu (C.S.); 2 Department of Surgery, Hennepin County Medical Center, Minneapolis, MN 55455, USA; 3 Division of Colon and Rectal Surgery, Department of Surgery, University of Minnesota, 420 Delaware St. SE, Minneapolis, MN 55455, USA; hnalluri@umn.edu (H.N.-B.); goffr002@umn.edu (P.G.); gaert015@umn.edu (W.B.G.); 4 Granger Medical Center, 1250 E 3900 S #320, Salt Lake City, UT 84124, USA; 5 School of Public Health, University of Minnesota, Minneapolis, MN 55455, USA; abbyj@umn.edu; 6 BioTechnology Institute, University of Minnesota, St. Paul, MN 55455, USA

**Keywords:** antibiotics, bowel preparation, gut microbiota, colon surgery, colonoscopy, IgA, short-chain fatty acids

## Abstract

The gut microbiome is essential for gut health, immune regulation, and metabolism, but pathogenic bacteria like *Enterococcus* and *Streptococcus* can disrupt these processes, increasing infection risk after colorectal surgery. Prior studies show that intravenous antibiotics and surgical bowel preparation (SBP, including mechanical preparation with oral antibiotics) significantly disrupt the gut microbiota, potentially delaying postoperative recovery. However, the effects of surgical indication (e.g., diagnosis) and operation type on gut microbiome composition and function remain unclear. This study examines how SBP, resectional and non-resectional surgery, and underlying diagnoses shape the postoperative gut microbiome and microbial recovery. Methods: Fecal samples were collected from patients undergoing colonoscopy (*n* = 30), non-resectional (ventral mesh rectopexy, transanal surgery; *n* = 25), or resectional surgery with primary anastomosis (*n* = 26) at baseline, intraoperatively, and on postoperative days (POD) 10, 30, and 180. Microbial diversity was assessed through 16S rRNA sequencing, and short-chain fatty acid (SCFA) levels were measured to evaluate functional changes. Results: Alpha diversity (Shannon indices) decreased across all groups, recovering by POD10 in colonoscopy patients and by POD180 in non-resectional and resectional cohorts. Beta diversity (community composition) also returned to baseline by POD10 in colonoscopy patients and POD180 in non-resectional patients, but the resectional cohort did not fully recover (*p* < 0.001). Both surgical cohorts showed substantial losses of commensal bacteria through POD30, with notable increases in Streptococcus in resectional patients (*p* < 0.0001) and Enterococcus in both surgical cohorts (*p* < 0.0001). Functionally, only the resectional cohort experienced significant reductions in SCFA levels (*p* < 0.015) relative to baseline levels. Diagnosis minimally influenced long-term microbiota recovery, although cancer patients tended to have more stable microbiomes compared to patients with diverticulitis. Conclusions: These findings indicate that perioperative factors, especially surgical resection and SBP, significantly impact gut microbial recovery, with pathogenic bacteria persisting up to 6 months post-surgery.

## 1. Introduction

Intestinal diseases are a significant health burden, with rising prevalence linked to Western diets and an aging population [1]. Surgery is often required for conditions like diverticulitis, colorectal cancer, and inflammatory bowel disease [2,3,4]. Successful bowel resection with primary anastomosis is critical for recovery, yet only half of colon operations achieve a complication-free outcome, leaving room for improvement [5,6]. Delayed return of bowel function [7,8] and infections [9,10,11,12], including anastomotic leaks (AL) and surgical site infections (SSI), are major contributors to poor outcomes, affecting around 20% of operations, despite optimal technique. These complications increase morbidity, mortality, hospital stays, and healthcare costs [13,14]. Despite the need to improve patient outcomes, therapeutic progress in preventing these complications has been hindered by fundamental gaps in understanding their etiology.

The gut microbiome plays a crucial role in maintaining gut health, immune regulation, and metabolic functions [15]. Injured intestinal mucosa is characterized by a microenvironment of cellular migration, proliferation, and tissue remodeling [16]. Certain microbiota subpopulations preferentially colonize damaged mucosa, promoting epithelial wound healing [17]. However, pathogenic bacteria can interfere, increasing the risk of infectious complications. For example, *Enterococcus faecalis* has been mechanistically linked to AL via the production of matrix metalloproteinase 9, which degrades collagen [18]. Mice intestinally colonized with methicillin-resistant *Staphylococcus aureus* can develop SSI by the silent migration of bacteria to the wound without bacteremia [19]. The microbiota can also contribute to systemic inflammation through the translocation of microbial products like lipopolysaccharides across the intestinal barrier [20].

Colorectal surgery introduces unique challenges to the gut microbiome. Surgical bowel preparation (SBP), involving mechanical bowel preparation (MBP) and oral antibiotics (OAs), is administered in addition to intravenous (IV) antibiotics at the time of surgery to reduce pathogen load and minimize infection risk [12,21,22,23,24,25,26]. However, SBP leads to significant reductions in beneficial commensal bacteria, including short-chain fatty acid (SCFA)-producing species, which are critical for gut barrier integrity and wound healing [17,27,28,29]. Furthermore, patients undergoing resectional surgery face additional challenges from anatomical disruption, which may exacerbate microbial disturbances and delay recovery. Despite the growing recognition of the microbiome’s role in surgical outcomes, little is known about the distinct contributions of perioperative factors, surgical anatomy, and baseline diagnoses (i.e., diverticulitis versus cancer) in shaping postoperative gut microbial composition.

Building on prior work [30,31], this study aims to explore the relative contributions of SBP, resectional versus non-resectional surgery, and underlying diagnoses on postoperative gut microbial community. This study included a colonoscopy group to model MBP without surgical stress and two surgical groups receiving SBP and either non-resectional or resectional surgery to determine the impact of anatomic disruption. Specifically, we hypothesized that patients undergoing resectional surgery would experience more profound and prolonged disruptions in microbial diversity and community structure than colonoscopy or non-resectional surgery due to anatomical alterations. Additionally, we sought to determine whether baseline diagnoses of diverticulitis or colorectal cancer would lead to distinct patterns of microbial reconstitution, mainly given the higher prevalence of prior antibiotic exposure in diverticulitis patients. Finally, we investigated the functional implications of these microbiome changes by measuring SCFA levels, which are crucial for gut health and healing. By elucidating the interplay between perioperative factors and microbial reconstitution, this study attempted to provide a foundation for developing targeted strategies that minimize microbial disruption and promote faster microbial recovery, ultimately improving surgical outcomes.

## 2. Results

### 2.1. Study Cohorts

Fecal samples were obtained from patients who underwent colonoscopy (*n* = 30), resectional surgery (*n* = 26), or non-resectional surgery (*n* = 25). Demographics and clinical data are presented in Table 1.

All surgery patients received perioperative IV antibiotics, though the specific regimen varied based on patient tolerance and allergies (Table 2). Within each cohort, all IV antibiotic groups had similar baseline composition without differences in alpha or beta diversity. Similarly, no significant differences in microbiome composition were observed between samples collected on POD10 and POD30 across the various antibiotic groups within both the non-resectional and resectional cohorts. Compared to colonoscopy patients, individuals undergoing surgery were generally older, and those undergoing resectional procedures were more frequently male. Patients from all three cohorts were similar with regards to comorbidities.

### 2.2. Diversity and Community Composition Within Cohorts

**Figure 1:**
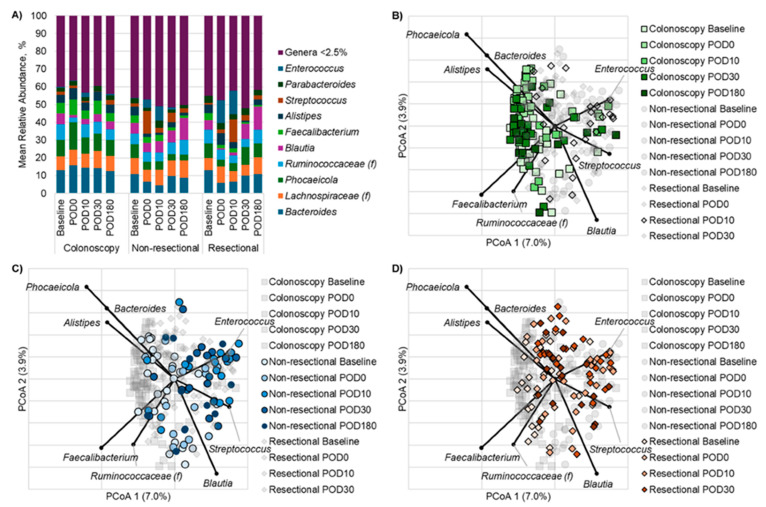
(**A**) Distribution of predominant genera (or most resolved classification) among patients receiving colonoscopy, non-resectional surgery, or resectional surgery over the course of the study; (f) denotes classification beyond family level was not possible. (**B**–**D**) Principal coordinate analysis of Bray–Curtis distances in colonoscopy (**B**), non-resectional (**C**), and resectional (**D**) cohorts at baseline, POD0, POD10, POD30, and POD180. Panels (**B**–**D**) highlight functionally distinct bacterial groups and are separated to emphasize specific trends in microbial disruption and recovery.

Among all cohorts, there were significant changes in diversity over time. In colonoscopy patients, the mean Shannon index (alpha diversity) decreased significantly from baseline to DOS (mean ± standard deviation: 3.68 ± 0.31 vs. 3.13 ± 0.47; Kruskal–Wallis *p* = 0.0002; Figure S1, Table S1) but quickly recovered by POD10 (3.55 ± 0.65). In contrast, the mean Shannon index of non-resectional surgery patients decreased significantly from baseline to POD30 (3.65 ± 0.25 vs. 3.11 ± 0.45; *p* < 0.0001; Figure S1) and did not recover until POD180 (3.39 ± 0.35). Similarly, the mean Shannon index of resectional surgery patients decreased significantly from baseline to POD30 (3.67 ± 0.41 vs. 2.94 ± 0.59; *p* = 0.001; Figure S1) and then recovered by POD180 (3.30 ± 0.27). Similar to alpha diversity, the community structure (beta diversity) of colonoscopy patients differed significantly from baseline to DOS (ANOSIM R = 0.28, *p* < 0.001; Figure 1) but recovered by POD10. In non-resectional surgery patients, community structures differed significantly between baseline and both POD10 and POD30 (R = 0.31, 0.35, respectively, *p* < 0.001, for both comparisons; Figure 1), showing more sustained community disruption. Interestingly, communities for resectional surgery patients differed significantly between baseline and all other time points (R = 0.36, 0.26, 0.24, 0.24, respectively, *p* < 0.001, for all comparisons; Figure 1).

**Figure 2:**
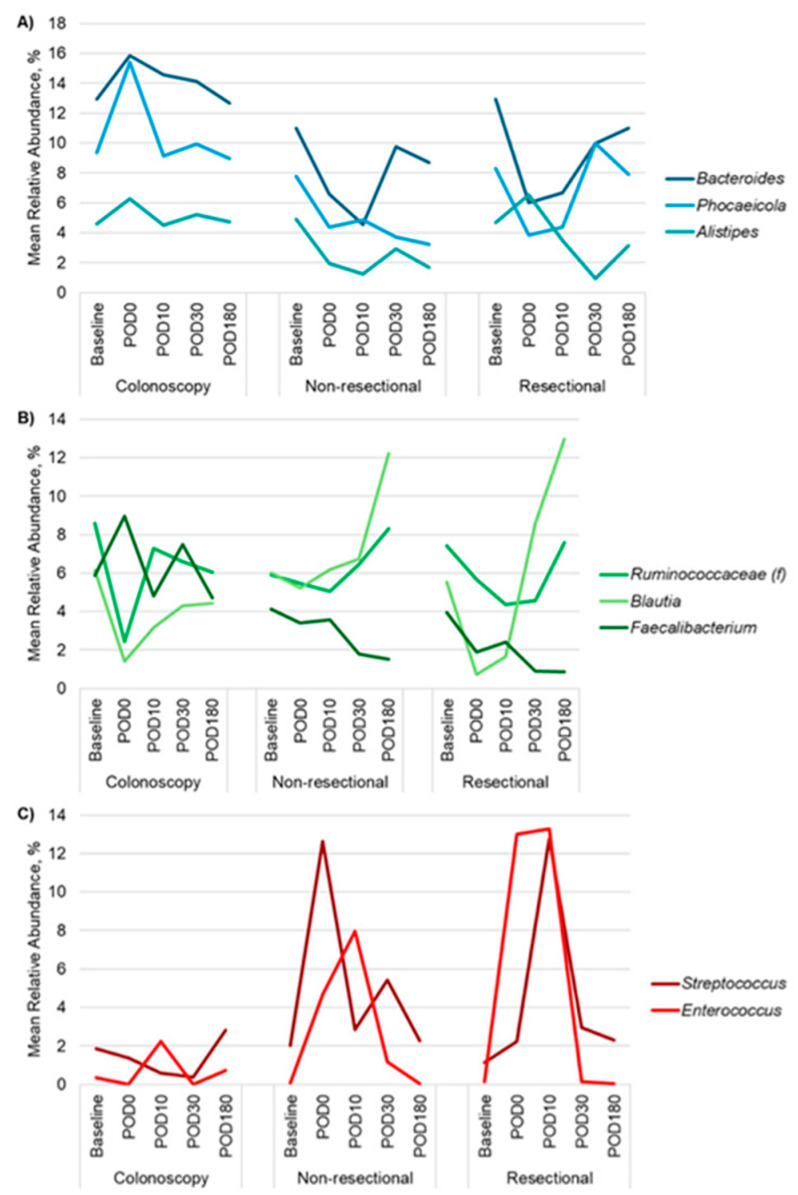
Abundances of predominant genera of patients prior to and up to POD180 following colonoscopy, non-resectional surgery, or resectional surgery. (**A**) Relative abundances of Bacteroides, Phocaeicola, and Alistipes. (**B**) Relative abundances of Ruminococcaceae, Blautia, and Faecalibacterium. (**C**) Relative abundances of Streptococcus and Enterococcus.

Several taxa exhibited significant changes in composition over time within the treatment cohorts. Among colonoscopy patients, *Ruminococcaceae*, *Blautia*, and *Streptococcus* significantly decreased from baseline to DOS (*p* < 0.0001, <0.0001, <0.001, respectively; Figure 2, Table S2), with all three taxa recovering by POD10. In the non-resectional cohort, beneficial commensals *Bacteroides*, *Phocaeicola*, and *Alistipes* decreased significantly from baseline to POD10 (*p* = 0.001, 0.002, <0.0001, respectively), while *Enterococcus* increased significantly during the same period (*p* < 0.0001) and remained elevated through POD30. By POD30, *Bacteroides* returned to baseline levels, while *Phocaeicola*, *Alistipes*, and *Enterococcus* did not return to baseline until POD180. In the resectional cohort, the relative abundances of *Bacteroides* and *Blautia* significantly decreased from baseline to POD10 (*p* = 0.003, 0.004, respectively), whereas *Streptococcus* and *Enterococcus* significantly increased over the same period (*p* = 0.0001 and 0.0004, respectively). By POD30, *Bacteroides*, *Blautia*, and *Streptococcus* had returned to baseline levels, though *Enterococcus* remained elevated until POD180.

### 2.3. Diversity and Community Composition Among Cohorts

Differences in microbial diversity were compared among the colonoscopy, non-resectional, and resectional cohorts throughout the surgery and recovery periods. On POD10, colonoscopy patients exhibited significantly greater Shannon indices (3.55 ± 0.65) compared to both non-resectional (2.73 ± 0.93) and resectional (2.70 ± 1.21) patients (*p* = 0.0002, 0.006, respectively; Table S1). Similarly, at POD180, colonoscopy patients maintained significantly greater Shannon indices (3.78 ± 0.41) compared to non-resectional (3.39 ± 0.35) and resectional (3.30 ± 0.27) patients (*p* = 0.011, 0.0005, respectively). Notably, there were no significant differences in Shannon indices between non-resectional and resectional patients at any time point.

When comparing across treatment cohorts at the same time points, several genera showed significant differences in relative abundance. On DOS, *Faecalibacterium* was more abundant in the colonoscopy group than in the resectional group (*p* < 0.0001; Figure 2, Table S2), and *Streptococcus* had significantly greater relative abundances in the non-resectional cohorts compared to the colonoscopy group (*p* < 0.0001). On POD10, the colonoscopy cohort exhibited a greater relative abundance of *Bacteroides* and lower levels of *Enterococcus* compared to the non-resectional cohort (*p* = 0.0002, 0.0001). The resectional cohort similarly had greater relative abundances of *Enterococcus* as well as *Streptococcus* relative to the colonoscopy group (*p* = 0.003 and 0.0001)**.** By POD30, the colonoscopy group had greater relative abundances of *Alistipes, Phocaeicola* and *Faecalibacterium* but lower levels of *Enterococcus* compared to both non-resectional and resectional patients (*p* ≤ 0.001). These findings suggest that while microbial diversity generally recovers over time across all cohorts, the resectional and non-resectional groups exhibit sustained shifts in the relative abundances of key genera such as *Streptococcus* and *Enterococcus*, particularly in the resectional cohort, which may reflect the differential impacts of surgical interventions on gut microbiota composition.

### 2.4. Diversity and Community Composition of Right Versus Left Colon Resections

To assess the relative impact of surgical anatomy on microbiome composition, we compared patients undergoing right-sided versus left-sided colon resections within the resectional cohort. There were no significant differences in the mean Shannon index between the two groups at baseline, POD10, or POD30. *Parabacteroides* was found in lower abundances in left resection patients compared to right resection patients on POD30 (*p* = 0.030, 0.009, respectively); however, this difference did not remain significant after applying a Bonferroni correction (corrected *α* = 0.001). Furthermore, no significant differences in overall community structure were observed between the left and right resection groups at any time point (R = 0.05, *p* > 0.05), suggesting that anatomical differences between left and right-sided resections are unlikely to significantly contribute to microbial communities relative to other perioperative factors.

### 2.5. Diversity and Community Composition Between Diverticulitis and Cancer

We next aimed to identify microbiome differences between patients with diverticulitis and those with colon cancer (including right- and left-sided together). On POD10, the mean Shannon index for colon cancer patients was significantly greater than that of diverticulitis patients (2.76 ± 0.25 vs. 1.70 ± 0.79; *p* < 0.0001; Table S3), though no significant differences were observed at other time points. Community structure differed significantly between diverticulitis and colon cancer patients on POD10 (R = 0.30, *p* = 0.001; Figure S2, Table S4); however, by POD30, the communities were similar. Among diverticulitis patients, community structure differed significantly from baseline to POD10 (R = 0.46, *p* < 0.001) and from POD10 to POD30 and POD180 (R = 0.47, 0.57, respectively, *p* < 0.001, for both comparisons). Among cancer patients, overall community structure did not differ at any time points.

**Figure 3:**
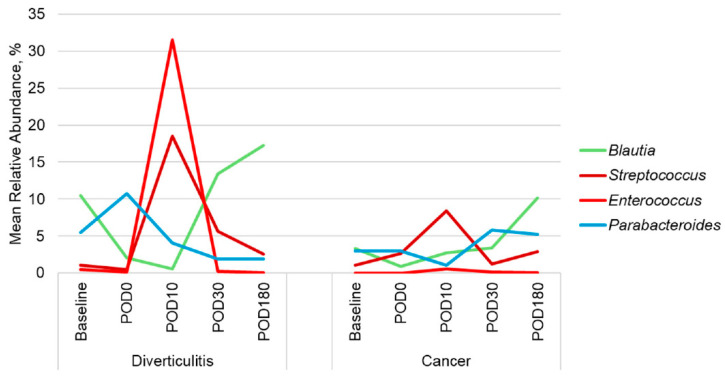
Distribution of predominant genera in diverticulitis and cancer patients who underwent resectional surgery at baseline, intraoperative (POD0), and postoperative (POD10, POD30, and POD180) time points.

In diverticulitis patients, the relative abundances of *Enterococcus* and *Streptococcus* significantly increased from baseline to POD10 (*p* = 0.002, 0.001, respectively; Figure 3, Table S4) before returning to baseline levels by POD30. In contrast, the relative abundance of the gut commensal genus *Blautia* decreased significantly from baseline to POD10 (*p* = 0.002) then returned to baseline by POD30. Among cancer patients, *Blautia* was the only genus that showed a significant change over time, with its relative abundance increasing from DOS to POD180 (*p* = 0.002). These results suggest that while microbiome diversity and community composition undergo significant shifts in diverticulitis patients during the early postoperative period, they tend to stabilize by POD30, whereas colon cancer patients exhibit a more stable microbial profile.

### 2.6. Two Resectional Patients with Anastomotic Leaks

Two patients experienced anastomotic leaks following resectional surgery. These patients were matched by age, BMI, and primary diagnosis to two non-leak patients. SourceTracker analysis of fecal samples on POD30 illustrated <0.1% and 3.2% similarity to baseline in leak patients and 54% and 72% similarity to baseline in non-leak patients, likely due to the prolonged course of antibiotics required at the time of AL diagnosis. There were no significant differences in relative abundances of genera, mean Shannon index, or community structure between the two leak patients and non-leak patients who underwent resectional surgery. These findings are limited by the study’s limited statistical power, given that the leak group consisted of only two patients.

### 2.7. Evaluation of Short-Chain Fatty Acids and IgA

Baseline and POD10 samples were analyzed for SCFA composition (Table 3).

Among POD10 samples, one sample in the resectional group had an SCFA concentration > 3 standard deviations above the mean and was excluded from statistical comparisons due to its extreme magnitude and inconsistency with expected postoperative SCFA levels based on both group trends and known physiological ranges. At baseline, concentrations of SCFAs were similar among groups, except for concentrations of isobutyrate and isovalerate, which were lower in the non-resectional cohort when compared to the resectional cohort (Tukey’s post hoc *p* = 0.013 and 0.046). In the resectional cohort but not the non-resectional group, all SCFA concentrations were significantly reduced from baseline to POD10 (*p* < 0.015). Concentrations of IgA normalized to total protein were significantly lower in the colonoscopy group compared to either surgery group (Table S5), but fecal IgA concentrations remained similar in all groups between baseline and POD10 samples.

**Figure 4:**
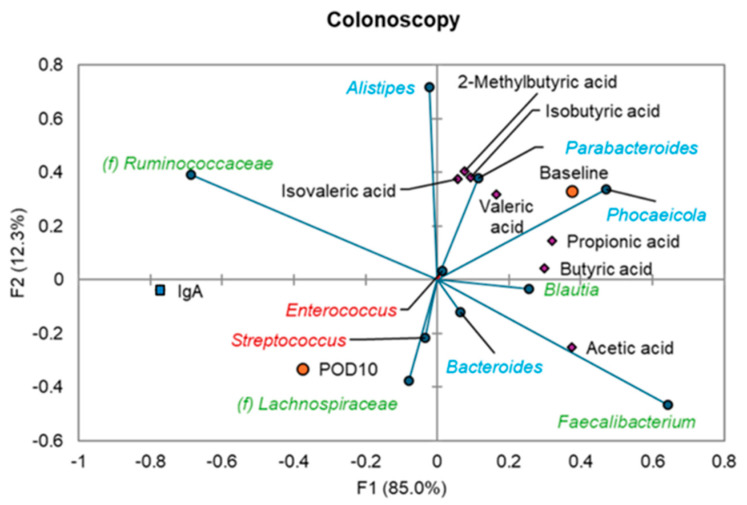
Canonical correspondence analysis relating genera, SCFAs, and IgA among samples from the colonoscopy cohort at baseline and POD10. Time points are shown as circles, SCFAs as diamonds, and IgA as a square. Taxa are colored by phyla: green—Bacillota; blue—Bacteroidota; red—Pseudomonadota.

In the colonoscopy cohort, multiple correlations were observed at baseline and POD10 between predominant genera and SCFA concentrations (Figure 4). Members of the Bacillota (e.g., *Ruminococcaceae, Lachnospiraceae*, and *Faecalibacterium*) and Bacteroidota (e.g., *Bacteroides, Phocaeicola,* and *Alistipes*) generally positively correlated with abundances of SCFAs (Spearman’s *ρ* = 0.439–0.779, *p* ≤ 0.048) at both baseline and POD10, with the exception of a negative correlation between *Phocaeicola* abundances and the acetic acid concentration at POD10 (*ρ* = −0.537, *p* = 0.034). At POD10, relative abundances of *Streptococcus* among colonoscopy patients negatively correlated with propionic acid and isovaleric acid (*ρ* = −0.553 and −0.533, *p* = 0.029 and 0.028). Similarly, *Enterococcus* negatively correlated with all SCFAs at POD10 (*ρ* = −0.548 to −0.677, *p* ≤ 0.030). *Phocaeicola* was the only genus among the colonoscopy patients that negatively correlated with fecal IgA concentrations at baseline (*ρ* = −0.440, *p* = 0.047).

Few correlations were observed among relative abundances of genera and SCFAs in the surgical cohorts. At baseline, *Parabacteroides* relative abundances were positively correlated with propionic acid in the non-resectional cohort (*ρ* = 0.567, *p* = 0.030), while *Faecalibacterium* abundances correlated with isobutyric acid in the resectional cohort (*ρ* = 0.520, *p* = 0.049). At POD10, *Lachnospiraceae*, *Ruminococcaceae*, *Blautia*, and *Alistipes* were correlated with valeric acid in the non-resectional cohort (*ρ* = 0.545, 0.780, 0.554, 0.635; *p* = 0.017, 0.0001, 0.015, 0.004, respectively), while *Blautia* was inversely correlated with fecal IgA concentrations (*ρ* = −0.473, *p* = 0.042).

## 3. Discussion

The gut microbiota plays a crucial role in influencing complications after intestinal surgery [18,19,32,33], yet the specific contributions of perioperative factors on gut microbiome recovery remain poorly understood. Building on our previous work [30,31], we examined the relative impact of MBP and SBP, type of colorectal operation (resectional versus non-resectional), and baseline diagnosis (diverticulitis versus cancer) on shaping postoperative microbiome composition. MBP, represented by the colonoscopy group, induced transient microbiome shifts, with recovery by POD10 [31]. SBP, surgical intervention, and underlying diagnosis significantly altered microbiome composition, with recovery in surgical cohorts taking up to six months. Given the increasing evidence supporting the enteral translocation of oral pathobionts during periods of gut dysbiosis, strategies to optimize postoperative microbial recovery are paramount to prevent post-surgical complications [34].

Limited knowledge exists regarding factors influencing gut microbiota recovery following surgery, particularly in the context of SBP administration. Considering the variability of surgical stress on the microbiome, we included a non-resectional surgical cohort to compare this impact in patients where anatomical continuity is preserved. Despite this, Shannon diversity indices showed a significant reduction for at least one month postoperatively before returning to baseline. In contrast, resectional patients undergoing partial colectomy exhibited more pronounced and prolonged changes in community structure, which remained altered for up to six months. Interestingly, within the resectional cohort, laterality had less influence on gut composition, as no significant differences were observed between patients undergoing right or left segmental colectomies. This pattern may reflect a more profound disruption of the microbial niche in resectional surgery, which is further explored below.

This prolonged disruption was most evident among patients undergoing resectional surgery, who exhibited more profound and sustained dysbiosis compared to those undergoing non-resectional procedures. This included depletion of key SCFA-producing taxa, such as *Blautia* and *Ruminococcaceae*, and prolonged elevation of facultative pathobionts like *Enterococcus*. We previously reported that *Blautia* and *Faecalibacterium*, both important SCFA producers, positively correlated with SCFA levels, while *Streptococcus* species, which primarily produce lactic acid, negatively correlated with SCFA production [31]. Increased levels of *Streptococcus* have been linked to dysbiosis and a decline in key butyrate-producing bacteria, including *Faecalibacterium* and *Blautia* [35]. Functionally, this correlated with a significant decrease in all measured SCFAs. We similarly observed an increase in *Streptococcus* in the resectional cohort and a subsequent drop in all SCFA concentrations following surgery. SCFA depletion is associated with gut and systemic inflammation, delayed wound healing, and gut barrier disruption [17,27].

In contrast, non-resectional patients demonstrated more rapid microbial recovery, with partial reconstitution of commensals by POD30. The greater disruption in the resectional group may be explained by several physiologic mechanisms. Ischemia-reperfusion injury during vessel ligation and bowel manipulation can damage mucosal integrity and alter the metabolic environment of the colon [36]. Surgical resection also disrupts enteric neural signaling and colonic transit, both of which influence luminal pH, oxygen tension, and nutrient availability [37,38]. These factors are critical for maintaining anaerobic microbial populations. Further, perioperative antibiotic ablation of microbiota may reduce niche exclusion dynamics allowing the translocation of oral pathobionts like *Streptococcus* [34]. Additionally, temporary or permanent changes in bowel continuity may result in increased luminal oxygen levels, which can favor the overgrowth of *Enterococcus* and *Streptococcus* and further delay the re-establishment of an anaerobic, SCFA-producing community [39]. These interrelated mechanisms likely contribute to the delayed microbial recovery observed in the resectional group and underscore that current perioperative practices may not sufficiently support mucosal healing, particularly in patients with anastomoses where gut recovery is critical to surgical success.

When comparing patients undergoing resection for diverticulitis versus colorectal cancer, we found distinct patterns in microbial recovery. Patients with diverticulitis exhibited a delayed return of commensal SCFA-producing taxa such as *Blautia* and *Faecalibacterium* [40], along with sustained elevations in *Streptococcus* and *Enterococcus* through POD30. In contrast, cancer patients showed more rapid suppression of pathobionts and the partial reconstitution of commensals by POD180. These differences likely reflect variation in the baseline microbial landscape: diverticulitis patients often have a history of chronic mucosal inflammation, recurrent antibiotic exposure, and altered colonic motility that may impair microbiome resilience [41,42]. In contrast, tumor-associated dysbiosis, such as *Fusobacterium* and *Parabacteroides*, may be partially eliminated during oncologic resection, allowing for different reassembly trajectories [43,44,45,46,47,48,49,50,51]. *Blautia* recovery by POD180 was more robust in cancer patients, while *Enterococcus* levels remained elevated longer in diverticulitis patients. These dynamics suggest ecological competition, in which the re-emergence of beneficial anaerobes may suppress the persistence of opportunistic pathobionts. These findings support the idea that underlying disease etiology contributes to the pace and pattern of microbial recovery and may have implications for tailoring microbiota-targeted therapies after surgery.

Despite the comprehensive nature of this study, several limitations should be considered. The overall sample size for each subgroup may limit the statistical power of our findings, particularly for specific comparisons, such as cancer versus diverticulitis patients and cancer location. Larger studies are required to confirm these findings and further explore subgroup-specific effects given that these comparisons were exploratory. While we excluded patients who received systemic antibiotics within 30 days prior to enrollment to reduce baseline microbiome variability, we did not account for more remote or cumulative exposures (e.g., multiple antibiotic courses in the preceding year). These unmeasured exposures may have influenced microbial diversity and recovery trajectories [41]. Future studies should also incorporate multiomics approaches to resolve microbial functional pathways and better characterize host–microbiome interactions. These tools will allow for a more comprehensive understanding of how perioperative interventions affect microbial metabolism, antimicrobial resistance, and mucosal immune responses. Furthermore, our study was conducted at a single medical center, which may limit generalizability given potential differences in surgical techniques, bowel preparation protocols, and perioperative practices. Future multicenter studies are needed to validate our results. Although SCFA levels were quantified as functional markers, other metabolites such as bile acids were not assessed and could provide additional insight. Our study accounted for key variables such as age, sex, BMI, and comorbidities, but other factors like diet, lifestyle, and additional perioperative care interventions were not controlled for and may have influenced gut microbiome composition and recovery. Lastly, alternative normalization methods (e.g., cumulative sum scaling and variance-stabilizing transformation) are increasingly used to address uneven sequencing depth. Because all included samples achieved >98% Good’s coverage at our rarefaction threshold, biological inferences are unlikely to differ with these approaches, consistent with prior work [52,53]. We therefore retained rarefaction as our primary strategy while acknowledging these alternatives as complementary options.

Taken together, these findings highlight the need for personalized perioperative strategies that preserve microbial resilience, particularly in patients undergoing resectional surgery. Future research should focus on microbiome restoration protocols and long-term follow-up to better understand how to optimize recovery and reduce postoperative complications through targeted microbial support.

## 4. Materials and Methods

### 4.1. Study Design

Eighty-one adult patients were recruited from March 2019 to April 2024 and underwent colonoscopy, elective resectional, or elective non-resectional colorectal surgery at the University of Minnesota Medical Center. This was a prospective observational cohort study. Participants were enrolled and followed longitudinally at predefined postoperative time points. Fecal samples were obtained pre-procedurally or preoperatively (within 30 days), intra-procedurally or on the day of surgery (DOS), post-procedurally or postoperatively within 10 days (POD10), within 3–6 weeks (POD30), and at 6 months (POD180).

Colonoscopy patients underwent MBP alone, while surgery patients underwent SBP composed of MBP with OAs and perioperative IV antibiotics. MBP included either MiraLAX (238 g) plus magnesium citrate (296 mL), GoLYTELY, or MiraLAX plus Gatorade. Oral antibiotics included neomycin (1 g q12 h for 2 doses) plus metronidazole (500 mg q6 h for 3 doses) given with prophylactic Zofran (4 mg q6 h for 3 doses) as an anti-nausea measure. All patients undergoing surgery received perioperative antibiotic prophylaxis in accordance with SCIP guidelines [54] and were initiated 1–2 h before incision and continued selectively for up to 24 h postoperative. Regimens included ciprofloxacin (400 mg q12 h) plus metronidazole (500 mg q8 h), cefazolin (2 g if <120 kg or 3 g if ≥120 kg) plus metronidazole, ertapenem (1 g daily), and cefotetan (1–2 g q12 h) at the discretion of the surgeon. Postoperative diet consisted of liquids until evidence of return of bowel function. The choice of laparoscopic versus open surgery was at the discretion of the attending colorectal surgeon, consistent with standard clinical practice and patient anatomy/pathology. Patients were advanced to a low-fiber diet for four weeks, after which dietary restrictions were lifted and a high-fiber diet was recommended. Exclusion criteria included history of organ transplant, active immunosuppression, chronic steroid use, any chemotherapy 12 months before procedure, history of intra-abdominal or pelvic radiation, inflammatory bowel disease, previous colorectal resection, planned diverting ileostomy, and pregnancy. Patients were excluded if they had received systemic antibiotic therapy within 30 days prior to enrollment, except for antibiotics administered as part of standard perioperative prophylaxis. This criterion was applied to reduce potential variability in baseline microbiota composition related to recent antimicrobial exposure. Human fecal specimens were obtained under approval by the Institutional Review Board of the University of Minnesota. Informed written consent was obtained from each study participant before enrollment.

### 4.2. Sample Collection

Fecal samples were collected by patients in single-use specimen pans and transferred to 30 mL polystyrene fecal specimen containers (Globe Scientific, Inc., Paramus, NJ, USA), as previously described [31]. In brief, patients stored samples in their home freezers prior to their clinic, procedure, or surgery visit. Intra-procedural or intraoperative samples were collected by the treating surgeon via endoscopic suction of colonic effluent. Specimens were placed in 30 mL polystyrene containers and immediately transferred to a −80 °C freezer until processing.

### 4.3. DNA Extraction and Sequencing

DNA was extracted from 250 mg thawed fecal samples using the DNeasy PowerSoil Pro kit (QIAGEN, Hilden, Germany) on the automated QIAcube platform following the inhibitor removal technology (IRT) protocol. The V4 hypervariable region of the 16S rRNA gene was amplified using the 515F/806R primer set [55] by the University of Minnesota Genomics Center (UMGC, Minneapolis, MN, USA), as previously described [56]. Paired-end, dual-indexed sequencing at a read length of 300 nucleotides (nt) was performed on the Illumina MiSeq platform (Illumina, Inc., San Diego, CA, USA) by UMGC [56]. Negative sterile water controls were included in all sequencing runs and did not produce amplicons. Raw data were stored in the Sequence Read Archive under BioProject accession number SRP250717.

### 4.4. Bioinformatics

Amplicon sequence data were processed and analyzed using mothur software ver. 1.41.1 [57] with our previously published pipeline for quality screening and taxonomic annotation [58]. Briefly, reads were paired-end joined, quality trimmed, and aligned against the SILVA database ver. 138 [59]. Sequences were further cleaned using a 2% pre-clustering step [60], and chimeric sequences were identified and removed using UCHIMER ver. 4.2.40 [61]. Operational taxonomic units (OTUs) were binned at 99% similarity using the OptiClust algorithm [62], and taxonomic assignment was performed against the Ribosomal Database Project ver. 16 [63]. Across all samples, the lowest sequencing depth was 10 reads, and the highest was 117,425 reads (median 19,826; interquartile range 10,046–28,870).

For unbiased statistical comparisons [64], samples were rarefied to 5000 sequence reads by random subsampling, and samples with fewer sequence reads were removed from the dataset. At a rarefaction depth of 5000 reads, 23 samples of 323 (7%) were excluded. A mean estimated Good’s coverage of 98.5 ± 1.3% and a mean of 176 ± 99 OTUs were observed among all samples. The number of fecal samples included in comparisons among sample groups at each time point after normalization by rarefaction are shown in Table S1.

SourceTracker was used with default parameters to assess the similarity of samples from leak and matched patients to their own preoperative microbiota [65]. This software uses a Bayesian inference approach to determine what percent of the community of sink samples (postoperative samples) is derived from source (preoperative) samples taking an OTU table as input.

### 4.5. Statistical Analysis

Alpha and beta diversity statistics were calculated in mothur. Alpha diversity was evaluated with the Shannon index [66], which accounts for richness and evenness. Beta diversity was calculated using Bray–Curtis distances [67] and was visualized by ordination using principal coordinate analysis (PCoA) [68]. Spearman correlations were calculated to determine abundant genera that were significantly associated with PCoA axis position using the corr.axes command in mothur. Spearman correlations were also performed to determine associations between relative abundances of genera and SCFAs and IgA concentrations. Canonical correspondence analysis (CCA) was used for multivariate analysis. Patient demographic and clinical data were compared using Chi-squared analysis for categorical variables and ANOVA test for continuous variables. Differences in diversity indices were determined using ANOVA with Tukey’s post hoc test. Community compositions at different time points were compared using analysis of similarity (ANOSIM) [69] with Bonferroni correction for pairwise comparisons. Analyses were conducted with XLSTAT (version 2020.2.3; Addinsoft, Belmont, MA, USA). All statistics were evaluated at *α* = 0.05.

## Figures and Tables

**Table 1:** Patient demographic and clinical data. Continuous data presented as mean ± standard deviation.

	Colonoscopy (*n* = 30)	Resectional (*n* = 26)	Non-Resectional (*n* = 25)	*p*-Value
Male sex, *n* (%)	14 (47)	18 (69)	7 (28)	0.011
Age (years)	52.8 ± 15.4	57.2 ± 11.0	62.9 ± 12.2	0.017
BMI (kg/m^2^)	26.5 ± 5.1	28.9 ± 5.5	29.2 ± 7.2	0.178
Albumin	4.0 ± 0.4	3.9 ± 0.4	4.1 ± 0.5	0.164
Charlson comorbidity index	1.9 ± 2.3	2.6 ± 2.6	2.7 ± 2.0	0.423
**Smoking status, *n* (%)**				0.037
Active smoker	1 (3)	5 (19)	5 (20)	-
Former smoker	7 (23)	9 (35)	11 (44)	-
Non-smoker	22 (73)	12 (46)	9 (36)	-
Chronic narcotics, *n* (%)	0 (0)	1 (4)	1 (4)	0.390
**History of diabetes mellitus, *n* (%)**	0 (0)	5 (19)	4 (16)	0.011
Metformin	0 (0)	4 (15)	3 (12)	-
**Preoperative bowel prep, *n* (%)**				<0.0001
MiraLAX and Mag Citrate	15 (50)	25 (96)	10 (40)	-
GoLYTELY	13 (43)	1 (4)	0 (0)	-
MiraLAX and Gatorade	2 (7)	0 (0)	14 (56)	
Fleet enemas	0 (0)	0 (0)	1 (4)	-
**Postoperative complications, *n* (%)**				0.124
None	30 (100)	20 (77)	23 (92)	-
Anastomotic leak/sepsis	-	2 (8)	-	-
Urinary tract infection	-	2 (8)	1 (4)	-
Surgical site infection	-	-	-	-
Deep organ space infection	-	1 (4)	-	-
Pneumonia	-	-	-	-
Ileus requiring nasogastric tube	-	1 (4)	-	-
Anemia requiring transfusion	-	-	-	-
Acute renal failure	-	1 (4)	-	-
Deep venous thrombosis	-	-	-	-
**30-day hospital readmission, *n* (%)**	1 (3)	2 (8)	1 (4)	0.742

**Table 2:** Perioperative details and clinical history of surgery patients.

	Group B (Resectional) *n* (%)	Group C (Non-Resectional) *n* (%)
**Preoperative enteral antibiotics**		
Neomycin plus metronidazole	26 (100)	25 (100)
**Perioperative intravenous antibiotics**		
Ciprofloxacin plus metronidazole	10 (38)	7 (28)
Cefazolin plus metronidazole	7 (27)	8 (32)
Cefotetan	5 (19)	-
Ertapenem	4 (16)	10 (40)
**Operative procedure**		
Right colectomy	7 (27)	-
Left colectomy	1 (4)	-
Sigmoid colectomy	9 (35)	-
Sigmoid colectomy, diverting loop ileostomy	1 (4)	-
Low anterior resection	5 (19)	-
Ventral rectopexy	-	13 (52)
Transanal excision	-	12 (48)
History of colorectal cancer	12 (46)	12 (48)
**Pathology**		
Adenocarcinoma	11 (92)	8 (67)
Neuroendocrine tumor	1 (8)	-
Serrated adenoma with high-grade dysplasia	-	2 (16)
Tubulovillous adenoma with high-grade dysplasia	-	2 (16)
**Pathologic Stage**		
0	-	4 (33)
I	2 (17)	4 (33)
IIA	4 (33)	-
IIIA	3 (25)	-
IIIB	3 (30)	-
Not staged	-	4 (33)
**Diverticulitis History**		
Recurrent, uncomplicated	5 (50)	-
Complicated	5 (50)	-

**Table 3:** Fecal SCFA concentrations (µM/g) in patients undergoing colonoscopy, resectional, or non-resectional surgery at baseline and POD10.

Procedure	Time Point (n)	Acetate	Propionate	Isobutyrate	Butyrate	Isovalerate	Valerate	2-Methylbutyrate
Colonoscopy	Baseline (22)	23.19 (16.61) ^AB^	20.19 (10.81) ^B^	3.40 (2.22) ^AB^	17.52 (8.86) ^A^	2.32 (1.24) ^AB^	3.30 (1.59) ^A^	1.90 (1.15) ^A^
	POD10 (16)	23.37 (25.40) ^AB^	15.71 (10.25) ^AB^	3.29 (2.39) ^ABC^	15.26 (10.11) ^A^	2.05 (1.70) ^ABC^	2.04 (1.84) ^AB^	1.58 (1.41) ^AB^
Resection	Baseline (16)	26.41 (17.94) ^A^	25.68 (13.34) ^A^	3.54 (3.49) ^A^	18.89 (12.06) ^A^	3.40 (1.76) ^A^	3.70 (2.21) ^A^	2.18 (0.79) ^A^
	POD10 (14)	5.06 (4.90) ^B^	5.31 (4.81) ^B^	2.21 (4.23) ^B^	2.21 (4.23) ^B^	0.68 (1.17) ^C^	0.57 (0.79) ^C^	0.39 (0.69) ^C^
Non-resection	Baseline (16)	16.78 (13.96) ^AB^	15.65 (11.18) ^AB^	2.90 (2.89) ^BC^	12.06 (6.56) ^A^	1.66 (1.11) ^BC^	2.53 (1.85) ^BC^	1.16 (0.69) ^AB^
	POD10 (19)	17.83 (20.42) ^AB^	7.77 (8.57) ^B^	2.65 (2.98) ^BC^	9.35 (10.11) ^AB^	1.55 (1.68) ^BC^	0.71 (1.17) ^BC^	0.78 (1.33) ^BC^
Blanks	(5)	1.25 (0.12)	0.04 (0.01)	0.03 (0.01)	0.01 (0.01)	0.01 (0.01)	0.01 (0.01)	0.01 (0.01)
*p*-value		0.024	<0.0001	<0.001	<0.001	<0.0001	<0.0001	<0.0001

Concentrations are mean (SD) of SCFA (µM/g stool). Values sharing the same superscript letter do not differ significantly (Tukey’s post hoc test, *p* > 0.05). Values with different letters indicate statistically significant differences.

## Data Availability

The dataset supporting the conclusions of this article is available in the Sequence Read Archive repository under BioProject accession number SRP250717. It is available at https://www.ncbi.nlm.nih.gov/sra/?term=SRP250717 (accessed on 8 July 2025).

## Supplementary Materials

The following supporting information can be downloaded at: https://www.mdpi.com/article/10.3390/antibiotics14090881/s1, Table S1: Mean (±standard deviation) Good’s coverage, observed richness (Sobs), Shannon, and Chao1 indices for colonoscopy, non-resectional, and resectional patients over time. Table S2: Mean (±standard deviation) relative abundance for colonoscopy, non-resectional, and resectional patients over tim; Table S3: Mean (±standard deviation) Good’s coverage, observed richness (Sobs), Shannon, and Chao1 indices for diverticulitis and cancer patients who underwent resectional surgery over time; Table S4: Mean (±standard deviation) relative abundance for diverticulitis and cancer patients who underwent resectional surgery over time: Table S5: Percent fecal IgA concentrations relative to total protein at baseline and POD10 in colonoscopy, non-resectional surgery, and resectional surgery. Figure S1: Box plots of Shannon indices for (A) colonoscopy, (B) non-resectional surgery, and (C) resectional surgery patients at baseline, DOS, POD10, POD30, and POD180. Figure S2: (A) Predominant genera for diverticulitis and cancer patients who underwent resectional surgery at baseline, DOS, POD10, POD30, and POD180. (f) denotes classification beyond family-level was not possible. (B) Principal coordinate analysis of Bray-Curtis distances in diverticulitis and cancer patients who underwent resectional surgery at baseline, DOS, POD10, POD30, and POD180.
