# Supplementary material for: The Role of Surgical and Perioperative Factors in Shaping Gut Microbiome Recovery After Colorectal Surgery
Source: Antibiotics (Basel). 2025 Aug 31;14(9):881. doi: 10.3390/antibiotics14090881 (PMC12466354; doi:10.3390/antibiotics14090881)

Supplementary Materials

Table S1. Mean (± standard deviation) Good's coverage, observed richness (S_{obs}), Shannon, and Chao1 indices for colonoscopy, non-resectional, and resectional patients over time.

Cohort	Time point	n	Coverage, %	Sobs	Shannon Index	Chao1 Index
Colonoscopy	Baseline	29	98.49 ± 1.11	193 ± 71	3.68 ± 0.31	384 ± 426
	POD0	23	98.64 ± 1.34	158 ± 90	3.13 ± 0.47	349 ± 448
	POD10	18	98.42 ± 1.07	191 ± 78	3.55 ± 0.65	382 ± 321
	POD30	20	98.07 ± 1.56	223 ± 92	3.76 ± 0.35	539 ± 708
	POD180	14	97.78 ± 1.65	233 ± 100	3.78 ± 0.41	564 ± 526
Non-resectional	Baseline	25	98.06 ± 1.45	209 ± 84	3.65 ± 0.25	591 ± 675
	POD0	14	98.83 ± 0.32	165 ± 35	3.17 ± 0.64	533 ± 1098
	POD10	20	98.85 ± 1.75	118 ± 117	2.76 ± 0.93	368 ± 727
	POD30	16	99.22 ± 0.33	121 ± 39	3.11 ± 0.45	174 ± 63
	POD180	12	98.97 ± 0.45	147 ± 44	3.39 ± 0.35	221 ± 83
Resectional	Baseline	26	97.87 ± 1.70	217 ± 104	3.67 ± 0.41	727 ± 926
	POD0	15	98.05 ± 1.73	201 ± 122	3.03 ± 1.48	553 ± 555
	POD10	24	98.63 ± 0.78	172 ± 81	2.70 ± 1.21	278 ± 163
	POD30	23	99.03 ± 0.47	123 ± 36	2.94 ± 0.59	222 ± 119
	POD180	18	98.73 ± 0.78	157 ± 43	3.30 ± 0.27	318 ± 256

Table S2: Mean (± standard deviation) relative abundance for colonoscopy, non-resectional, and resectional patients over time

Cohort	Time point	*Bacteroides*	*Phocaeicola*	*Alistipes*	*Ruminococcaceae (f)*	*Blautia*	*Faecalibacterium*	*Streptococcus*	*Enterococcus*
Colonoscopy	Baseline	12.94 ± 8.18	9.36 ± 5.74	4.60 ± 4.15	8.59 ± 5.97	6.16 ± 4.97	5.89 ± 3.88	1.86 ± 4.20	0.36 ± 1.93
	POD0	15.84 ± 10.84	15.41 ± 12.93	6.27 ± 4.61	2.43 ± 1.69	1.40 ± 1.18	8.97 ± 6.20	1.37 ± 5.85	0.01 ± 0.03
	POD10	14.54 ± 10.08	9.15 ± 6.86	4.52 ± 3.97	7.29 ± 5.98	3.15 ± 2.77	4.80 ± 4.06	0.59 ± 0.78	2.23 ± 7.05
	POD30	14.13 ± 9.85	9.92 ± 5.36	5.21 ± 4.32	6.59 ± 4.48	4.30 ± 5.30	7.47 ± 4.81	0.36 ± 0.43	0.00 ± 0.01
	POD180	12.66 ± 9.25	8.95 ± 5.56	4.72 ± 2.97	6.06 ± 3.46	4.42 ± 4.25	4.71 ± 3.63	2.81 ± 6.87	0.71 ± 2.64
Non-resectional	Baseline	10.99 ± 8.39	7.78 ± 5.61	4.90 ± 4.76	5.89 ± 4.36	6.02 ± 6.38	4.13 ± 4.54	2.01 ± 3.02	0.07 ± 0.2
	POD0	6.59 ± 7.42	4.37 ± 6.55	1.95 ± 2.50	5.46 ± 4.57	5.21 ± 7.27	3.41 ± 4.89	12.64 ± 16.69	4.66 ± 17.41
	POD10	4.55 ± 6.88	4.84 ± 8.59	1.25 ± 2.90	5.07 ± 5.93	6.20 ± 9.75	3.59 ± 6.05	2.85 ± 3.25	7.96 ± 14.13
	POD30	9.75 ± 11.35	3.70 ± 6.77	2.93 ± 5.73	6.46 ± 8.30	6.73 ± 6.50	1.78 ± 3.31	5.43 ± 14.73	1.17 ± 3.77
	POD180	8.71 ± 11.07	3.25 ± 4.75	1.70 ± 3.01	8.32 ± 8.80	12.21 ± 11.8	1.52 ± 3.22	2.27 ± 3.24	0.03 ± 0.08
Resectional	Baseline	12.93 ± 7.61	8.29 ± 6.67	4.70 ± 4.08	7.41 ± 5.43	5.55 ± 6.66	3.95 ± 3.54	1.13 ± 2.07	0.13 ± 0.63
	POD0	6.02 ± 6.55	3.85 ± 4.56	6.54 ± 6.61	5.68 ± 5.87	0.73 ± 1.19	1.88 ± 3.77	2.23 ± 3.13	13.00 ± 34.25
	POD10	6.65 ± 9.16	4.38 ± 6.32	3.47 ± 4.74	4.35 ± 4.37	1.66 ± 2.06	2.42 ± 3.98	12.76 ± 14.98	13.29 ± 26.23
	POD30	9.98 ± 9.60	9.96 ± 12.05	0.95 ± 2.12	4.56 ± 5.30	8.59 ± 9.98	0.90 ± 2.86	2.96 ± 4.06	0.14 ± 0.25
	POD180	11.01 ± 11.32	7.89 ± 8.35	3.14 ± 7.02	7.59 ± 6.90	12.98 ± 10.88	0.86 ± 1.76	2.29 ± 2.40	0.05 ± 0.11

Table S3. Mean (± standard deviation) Good's coverage, observed richness (S_{obs}), Shannon, and Chao1 indices for diverticulitis and cancer patients who underwent resectional surgery over time

Cohort	Time point	n	Coverage, %	Sobs	Shannon Index	Chao1 Index
Diverticulitis	Baseline	7	98.95 ± 0.44	148 ± 55	3.35 ± 0.41	234 ± 97
	POD0	2	97.30 ± 2.42	246 ± 146	3.68 ± 0.44	1099 ± 1274
	POD10	10	98.96 ± 0.65	114 ± 51	1.70 ± 0.79	193 ± 116
	POD30	9	99.00 ± 0.40	144 ± 33	3.32 ± 0.34	224 ± 98
	POD180	9	99.16 ± 0.39	121 ± 38	3.17 ± 0.37	196 ± 85
Cancer	Baseline	9	97.23 ± 1.75	254 ± 90	3.76 ± 0.19	1143 ± 1268
	POD0	6	97.65 ± 2.11	236 ± 141	3.16 ± 1.45	582 ± 424
	POD10	7	99.02 ± 0.29	122 ± 31	2.76 ± 0.76	215 ± 67
	POD30	9	98.09 ± 0.53	247 ± 45	3.67 ± 0.25	388 ± 99
	POD180	6	98.87 ± 0.75	146 ± 34	3.26 ± 0.25	273 ± 204

Table S4. Mean (± standard deviation) relative abundance for diverticulitis and cancer patients who underwent resectional surgery over time

Cohort	Time point	*Blautia*	*Streptococcus*	*Enterococcus*	*Parabacteroides*
Diverticulitis	Baseline	10.51 ± 10.89	1.01 ± 1.46	0.46 ± 1.21	5.46 ± 5.16
	POD0	2.04 ± 1.78	0.48 ± 0.47	0.11 ± 0.16	10.75 ± 4.85
	POD10	0.56 ± 0.71	18.49 ± 18.88	31.52 ± 33.54	4.05 ± 8.17
	POD30	13.45 ± 13.22	5.64 ± 2.19	0.24 ± 0.07	1.85 ± 2.36
	POD180	17.26 ± 11.57	2.53 ± 5.25	0.04 ± 0.36	1.90 ± 4.51
Cancer	Baseline	3.28 ± 2.37	1.09 ± 1.31	0.00 ± 0.00	2.97 ± 2.82
	POD0	0.92 ± 1.43	2.62 ± 2.87	0.00 ± 0.00	3.01 ± 3.22
	POD10	2.75 ± 2.82	8.38 ± 7.31	0.53 ± 1.37	1.06 ± 1.48
	POD30	3.42 ± 4.77	1.23 ± 1.93	0.11 ± 0.14	5.79 ± 8.23
	POD180	10.18 ± 6.57	2.91 ± 3.01	0.08 ± 0.18	5.23 ± 6.82

Table S5. Percent fecal IgA concentrations relative to total protein at baseline and POD10 in colonoscopy, non-resectional surgery, and resectional surgery.

Procedure	Time Point (n)	Mean, %
Colonoscopy	Baseline (15)	0.047^{B}
	POD10 (16)	0.119^{B}
Non-resection	Baseline (15)	5.69^{A}
	POD10 (18)	3.36^{AB}
Resection	Baseline (14)	3.67^{AB}
	POD10 (22)	3.71^{AB}

^{AB}Values sharing the same letter do not differ significantly by Tukey's *post-hoc* test ($P > 0.05$).

Figure S1. Box plots of Shannon indices for **(A)** colonoscopy, **(B)** non-resectional surgery, and **(C)** resectional surgery patients at baseline, DOS, POD10, POD30, and POD180.

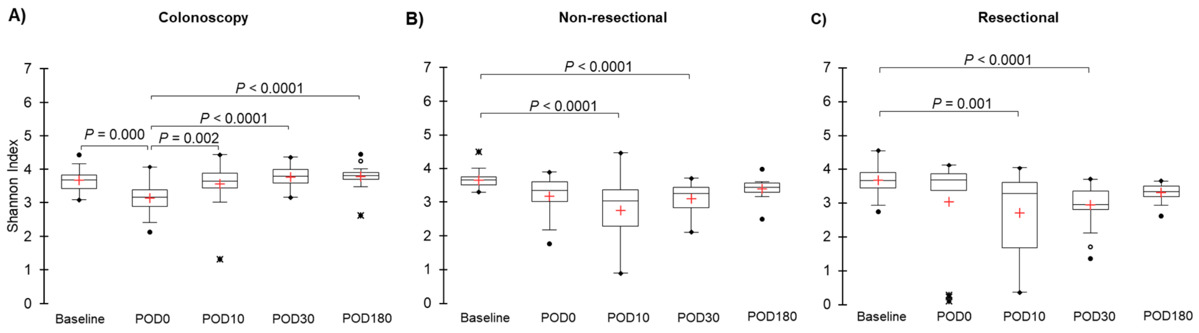

Figure S2. **(A)** Predominant genera for diverticulitis and cancer patients who underwent resectional surgery at baseline, DOS, POD10, POD30, and POD180. (*f*) denotes classification beyond family-level was not possible. **(B)** Principal coordinate analysis of Bray-Curtis distances in diverticulitis and cancer patients who underwent resectional surgery at baseline, DOS, POD10, POD30, and POD180.

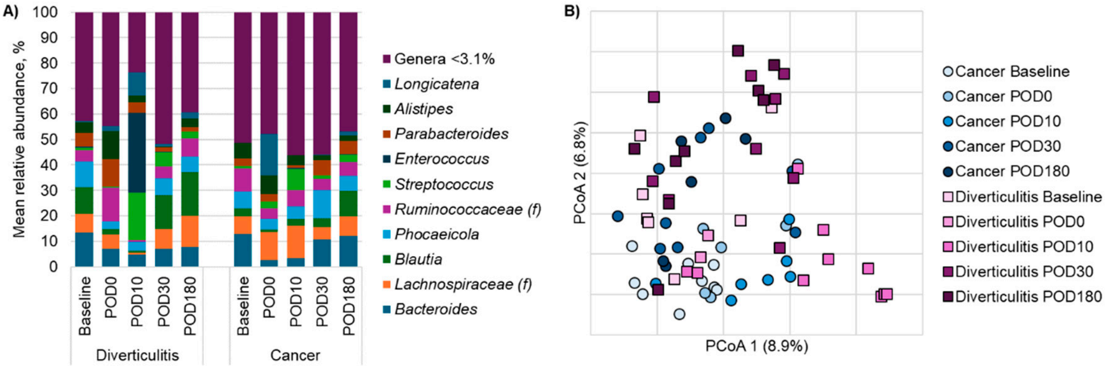

Supplement: Supplementary file 1 [file antibiotics-14-00881-s001.zip › antibiotics-3778987-supplementary.pdf]
